# Improvement of Liquid Fructose-Induced Adipose Tissue Insulin Resistance by Ginger Treatment in Rats Is Associated with Suppression of Adipose Macrophage-Related Proinflammatory Cytokines

**DOI:** 10.1155/2013/590376

**Published:** 2013-02-21

**Authors:** Jianwei Wang, Huanqing Gao, Dazhi Ke, Guowei Zuo, Yifan Yang, Johji Yamahara, Yuhao Li

**Affiliations:** ^1^Faculty of Basic Medical Sciences, Chongqing Medical University, Chongqing 400016, China; ^2^The Second Affiliated Hospital, Chongqing Medical University, Chongqing 400010, China; ^3^College of Laboratory Medicine, Chongqing Medical University, Chongqing 400016, China; ^4^Endocrinology and Metabolism Group, Sydney Institute of Health Sciences, Sydney, NSW 2000, Australia; ^5^Pharmafood Institute, Kyoto 602-8136, Japan

## Abstract

Adipose tissue insulin resistance (Adipo-IR) results in excessive release of free fatty acids from adipose tissue, which plays a key role in the development of “lipotoxicity.” Therefore, amelioration of Adipo-IR may benefit the treatment of other metabolic abnormalities. Here we found that treatment with the alcoholic extract of ginger (50 mg/kg/day, by oral gavage) for five weeks attenuated liquid fructose-induced hyperinsulinemia and an increase in the homeostasis model assessment of insulin resistance (HOMA-IR) index in rats. More importantly, ginger reversed the increases in the Adipo-IR index and plasma nonesterified fatty acid concentrations during the oral glucose tolerance test assessment. Adipose gene/protein expression profiles revealed that ginger treatment suppressed CD68 and F4/80, two important macrophage accumulation markers. Consistently, the macrophage-associated cytokines tissue necrosis factor alpha and interleukin-6 were also downregulated. In contrast, insulin receptor substrate (IRS)-1, but not IRS-2, was upregulated. Moreover, monocyte chemotactic protein (MCP)-1 and its receptor chemokine (C-C motif) receptor-2 were also suppressed. Thus these results suggest that amelioration of fructose-induced Adipo-IR by ginger treatment in rats is associated with suppression of adipose macrophage-related proinflammatory cytokines.

## 1. Introduction

Insulin resistance is the thread that runs through many chronic afflictions of modern times: obesity, cardiovascular disease, and, most conspicuously, type 2 diabetes [1]. While hepatic and muscle insulin resistance plays important roles, an additional component, adipose tissue insulin resistance (Adipo-IR), is also a significant factor to systemic insulin resistance, especially to the development of obesity-related insulin resistance [2, 3]. Adipose tissue is increasingly recognized as a secretory organ that plays many important roles in homeostasis, of which energy expenditure and insulin sensitivity are included. Adipose tissue is understood to exert its effects through both paracrine and endocrine mechanisms. Adipose tissue is emerging as a key mediator of cardiometabolic disorders in the general population and of liver disease in nonalcoholic fatty liver disease, likely through the modulation of lipotoxic free fatty acid metabolism and of pro- and anti-inflammatory cytokine secretion [3, 4]. Recent evidence suggests that the severity of Adipo-IR is closely correlated with metabolic derangements and hepatic histological damage in patients with nonalcoholic steatohepatitis [4–7]. Treatment with the insulin-sensitizing agent pioglitazone that improves adipose tissue functions [8] decreased Adipo-IR, which was correlated with the decreases in hepatic fat accumulation and necroinflammation in patients with nonalcoholic steatohepatitis [5, 7, 9]. These findings suggest that amelioration of Adipo-IR may play an important role in the treatment of nonalcoholic fatty liver disease.

Strong evidence suggests that consumption of diets high in fructose results in fatty liver, hyperlipidemia, and insulin resistance [10–12]. After absorption, fructose is almost completely metabolized in the liver, where fructose increases de novo lipogenesis [12]. The intrahepatic effects of fructose overconsumption have been extensively addressed [12]. It is also well known that fructose overconsumption is associated with adiposity [10–12]. Recent findings in clinic suggest that, in adolescents, higher fructose consumption is associated with multiple markers of cardiometabolic risk, but it appears that these abnormalities are mediated by visceral obesity [13]. Unfortunately, we still know much less about the adverse effects of fructose overconsumption on adipose tissue functions.

Ginger (*Zingiber officinale* Roscoe, Zingiberaceae) is one of the most commonly used spices and medicinal plants around the world. It has been found that ginger treatment ameliorates fatty liver and hyperlipidemia in rats fed cholesterol-enriched diet [14] and high-fat diet [15]. Recently, we have also demonstrated that ginger treatment ameliorates fructose-induced metabolic abnormalities, such as fatty liver and hypertriglyceridemia in rats [16]. Further, the hepatic pathways have been suggested in the lipid-lowering effects [14–16]. In the present study, we investigated the effects of fructose overconsumption on adipose tissue insulin functions and the impact of ginger treatment in rats.

## 2. Materials and Methods

### 2.1. The Alcoholic Extract of Ginger

 The alcoholic extract of ginger was prepared and identified as described previously [16]. Briefly, 5 kg sliced ginger rhizomes including the skin were immersed in 5 L 95% ethanol with intermittent shaking for 24 h, then refluxed for 3 h by heating. The filtrate was evaporated under reduced pressure below 45°C. The residue (yield: 9.6%) was designated as an alcoholic extract. The extract was quantified by HPLC method to contain two representative components: [6]-gingerol and [6]-shogaol in concentrations of 4.4% and 1.1%, respectively.

### 2.2. Animals: Diet and Experimental Protocol

 All animal procedures were in accordance with the “Principles of laboratory animal care” (http://grants1.nih.gov/grants/olaw/references/phspol.htm) and were approved by the Animal Ethics Committee, Chongqing Medical University, China.

Male Sprague-Dawley rats weighing 210–230 g and the standard diet were supplied by the laboratory animal center, Chongqing Medical University, China. Rats were housed in a temperature-controlled facility (21 ± 1°C, 55 ± 5% relative humidity) with a 12 h light/dark cycle. Animals were allowed free access to water and the standard diet for at least 1 week prior to starting the experiments.

Given that sugar-sweetened nonalcoholic beverages, such as soft drinks, appear as the major source of fructose for the population aged 6–50 years [12], liquid fructose was used in the present study. In initial experiments, we noted that compared to vehicle, ginger treatment significantly increased fructose intake when the rats had free access to 10% fructose in drinking water. In order to exclude the influence of the difference in intake of fructose (the primary pathogenic factor in the development of the adverse metabolic effects in this model), we adjusted the fructose consumption in ginger-treated rats to that of fructose controls. 24 rats were divided into 4 groups (*n* = 6 per group, 2 rats/cage): (1) water control, free access to water; (2) fructose control, free access to 10% fructose solution (w/v, preparation every day); (3) fructose ginger 20 mg/kg; and (4) fructose ginger 50 mg/kg, in which the fructose consumption was adjusted (by regulating the concentration of fructose solution) daily to that in the fructose-control group on the previous day. There was no difference in body weight between the groups before treatments commenced. Animals in ginger-treated groups were administered ginger extract 20 and 50 mg/kg (oral gavage, once per day) for 5 weeks, respectively. The rats in water- and fructose-control groups received vehicle (5% Gum Arabic) alone. All rats had free access to the standard chow. The consumed chow and fructose solution were measured daily, and the intake of fructose was calculated. At the end of week 4, oral glucose tolerance test (OGTT) was performed. After rats were deprived of chow but still had free access to water or fructose solution for 14 h on day 35, animals were weighed and killed. Epididymal white adipose tissue (eWAT) was collected and weighed. Segments of eWAT were snap-frozen in liquid nitrogen and stored at −80°C for subsequent determination of gene expression.

### 2.3. OGTT

 After being fasted for 14 h with free access to water, all rats received a glucose solution (2 g/kg in 10 mL) by the oral route. Blood samples were collected prior to and 20, 60, and 120 min after administration of glucose solution for determination of plasma concentrations of glucose (kit from Kexin Institute of Biotechnology, Shanghai, China), insulin (kit from Morinaga Biochemical Industries, Tokyo, Japan), and nonesterified fatty acid (NEFA) (NEFA-C kit, Wako, Osaka, Japan) using enzymatic methods or by ELISA. Hepatic insulin sensitivity was expressed as the homeostasis model assessment of insulin resistance (HOMA-IR) index {[fasted insulin (*μ*IU/mL) × fasted glucose (mM)]/22.5} [6, 17]. Adipo-IR index was calculated as the following formula: [Adipo-IR index = fasted insulin (mmol/L) × fasted NEFA (pmol/L)] [4–6, 9]. 

### 2.4. Histological Examination

 A portion of eWAT was fixed with 10% formalin and embedded in paraffin. 10-micron sections were cut and stained with hematoxylin and eosin for examination of adipose tissue histology (IX-81, Olympus Corporation, Tokyo, Japan). The adipocyte cross-sectional area was measured using an ImageJ 1.43 analyzing system.

### 2.5. Real-Time PCR

 Total RNA was isolated from eWATs of individual rats using TRIzol (Takara, Dalian, China). cDNA was synthesized using M-MLV RTase cDNA Synthesis Kit (Takara, Dalian, China) according to the manufacturer's instructions. Real-time PCR was performed with the CFX 96 Real Time PCR Detection System (Bio-rad Laboratories Inc, Hercules, CA, USA) using the SYBR Premix Ex Taq II (Takara, Dalian, China). The sequences of primers are shown in Table 1. Gene expression in individual samples was determined in duplicate and was normalized against the reference *β*-actin. Expression in water-control rats was arbitrarily assigned a value of 1.

### 2.6. Data Analysis

 All results are expressed as means ± SEM. Data obtained from experiments with more than two groups of animals were analyzed by ANOVA using StatView and followed by Student-Newman-Keuls testing to locate the differences between groups. Data obtained from experiments with two groups of animals were analyzed by the Student's *t*-test. *P* < 0.05 was considered to be statistically significant.

## 3. Results

### 3.1. Effects of Fructose Consumption in Rats

Recently, we have demonstrated that fructose-overconsumption-induced fatty liver and hypertriglyceridemia in rats are improved by ginger treatment [16]. In the present study, we focused on adipose function-related variables.

**Figure 1:**
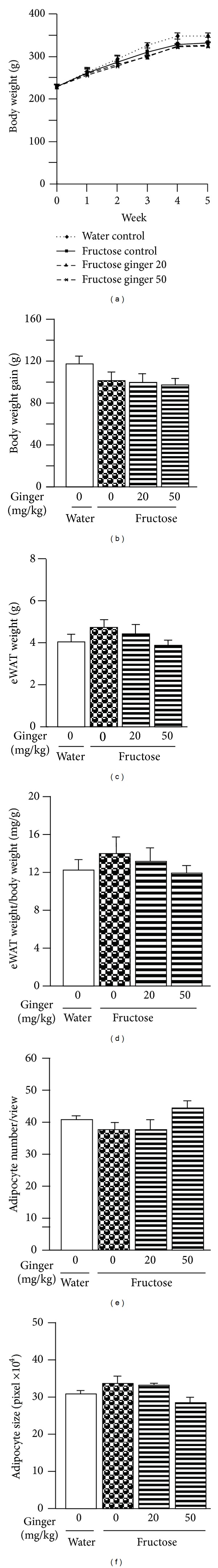
Body weight (a), body weight gain (b), epididymal white adipose tissue (eWAT) weight (c), ratio of eWAT weight to body weight (d), adipocyte number (e) and adipocyte size (f) in water-control and fructose-pair-fed rats. The fructose control rats had free access to 10% fructose in their drinking of water over 5 weeks, while the consumption of fructose in the ginger-(20 or 50 mg/kg) treated (by gavage daily) rats was adjusted to that of the fructose-control rats. Data are means ± SEM (*n* = 6 each group).

**Figure 2:**
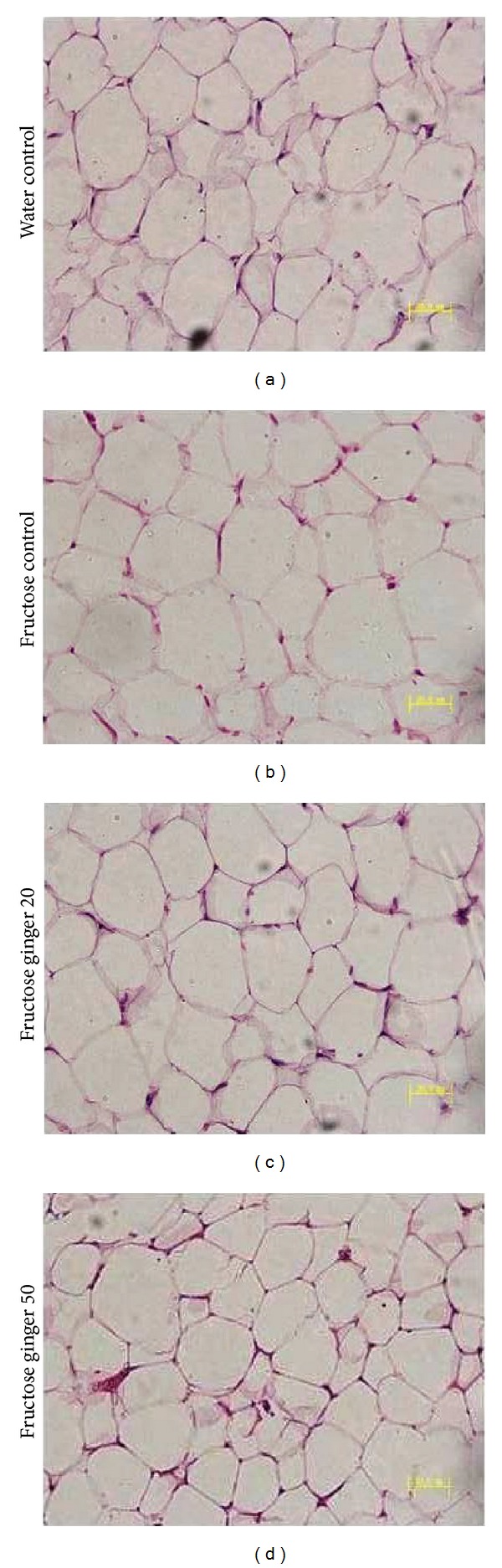
Representative images showing histology of eWAT (hematoxylin and eosin staining) in water-control or fructose-pair-fed rats (a)–(d). The fructose-control rats had free access to 10% fructose in their drinking water over 5 weeks, while the consumption of fructose in the ginger-(20 or 50 mg/kg) treated (by gavage daily) rats was adjusted to that of the fructose-control rats.

Five-week intake of 10% fructose solution decreased chow intake (water control: 1993.1 ± 100.0 versus fructose control: 1082.8 ± 75.6 g/2 rats/5 weeks, *P* < 0.05). Compared to water drinking, fructose feeding did not significantly affect body weights (Figure 1(a)) and body weight gain (Figure 1(b)). eWAT weight (Figure 1(c)), the ratio of eWAT weight to body weight (Figure 1(d)), and adipocyte size (Figures 1(f), 2(a) and 2(b)) had a trend to increase, whereas adipocyte number (Figure 1(e)) had a trend to decrease.

**Figure 3:**
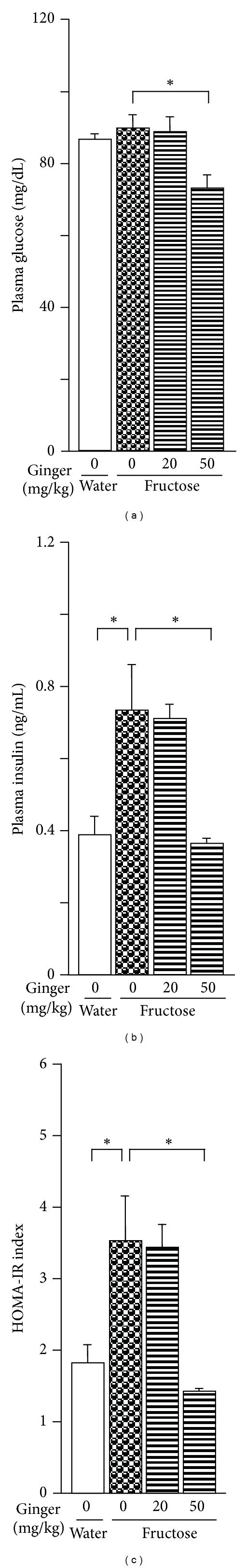
Plasma glucose (a) and insulin (b) concentrations at the baseline (fasted) and HOMA-IR index (c) in water-control and fructose-pair-fed rats. The fructose-control rats had free access to 10% fructose in their drinking water, while the consumption of fructose in the ginger-(20 or 50 mg/kg) treated (by gavage daily) rats was adjusted to that of the fructose-control rats. The variables were determined at the end of week 4. Data are means ± SEM (*n* = 6 each group). *P < 0.05.

Although fructose feeding did not alter basal plasma glucose concentration (Figure 3(a)), it significantly increased plasma insulin concentration under fasted condition (Figure 3(b)). The increase in insulin concentration resulted in an increase in the HOMA-IR index (Figure 3(c)).

**Figure 4:**
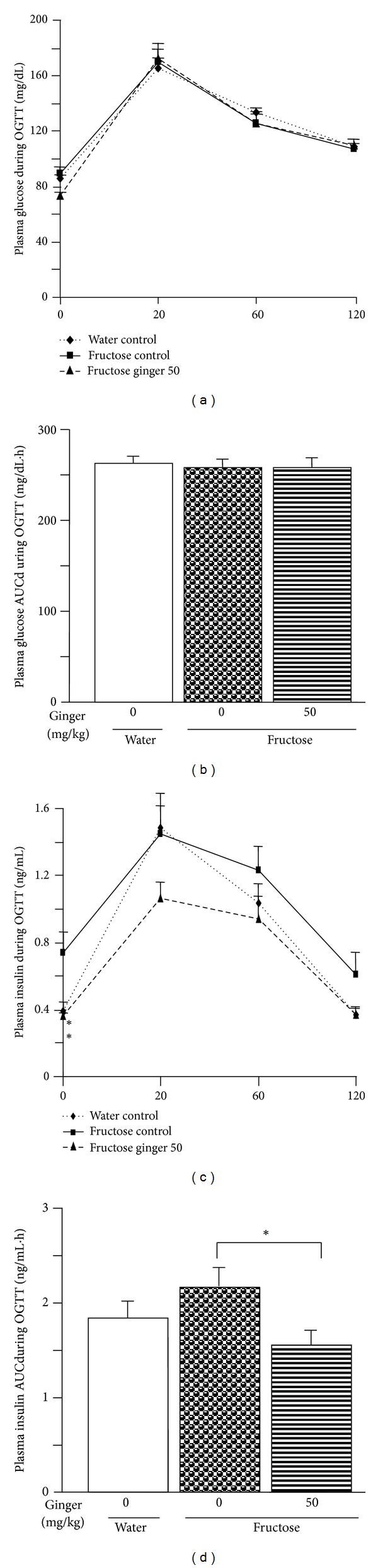
Plasma concentrations of glucose (a) and insulin (c) and their area under curve (AUC)s ((b) and (d)) during oral glucose tolerance test (OGTT, glucose: 2 g/kg) assessment in water-control and fructose-pair-fed rats. The fructose-control rats had free access to 10% fructose in their drinking water, while the consumption of fructose in the ginger-(20 and/or 50 mg/kg) treated (by gavage daily) rats was adjusted to that of the fructose-control rats. OGTT was performed at the end of week 4. Data are means ± SEM (*n* = 6 each group). *P < 0.05.

During the OGTT assessment, changes in plasma glucose (Figures 4(a) and 4(b)) and insulin (Figures 4(c) and 4(d)) concentrations were not significantly different from those of water-control group.

**Figure 5:**
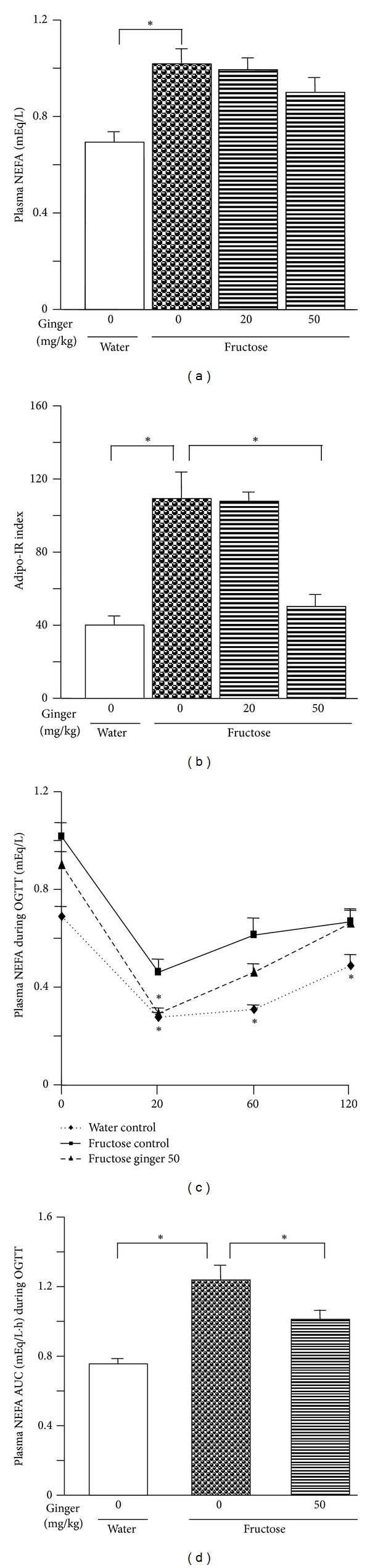
Plasma NEFA concentrations at the baseline (fasted) (a) and during oral glucose tolerance test (OGTT, glucose: 2 g/kg) assessment (c), the AUC of NEFA concentrations during OGTT (d), and the adipose tissue insulin resistance (Adipo-IR) index (b) in water-control and fructose-pair-fed rats. The fructose-control rats had free access to 10% fructose in their drinking of water, while the consumption of fructose in the ginger-(20 and/or 50 mg/kg) treated (by gavage daily) rats was adjusted to that of the fructose-control rats. OGTT was performed at the end of week 4. Data are means ± SEM (*n* = 6 each group). *P < 0.05.

Importantly, basal plasma NEFA concentrations (Figure 5(a)) and Adipo-IR index (Figure 5(b)) in fructose controls were higher compared to those in water controls. Furthermore, plasma NEFA concentrations (Figure 5(c)) and the NEFA AUC (Figure 5(d)) during the OGTT assessment were also increased.

### 3.2. Effects of Ginger Treatment in Fructose-Fed Rats

Fructose intake was uniform in fructose-control and fructose-ginger groups (fructose control: 845.4 ± 33.1; fructose ginger 20 mg/kg: 827.2 ± 13.6; fructose ginger 50 mg/kg: 831.8 ± 18.1 g/2 rats/5 weeks, *P* > 0.05). Ginger treatments did not significantly affect chow intake (fructose control: 1082.8 ± 75.6; fructose ginger 20 mg/kg: 1156.2 ± 29.1; fructose ginger 50 mg/kg: 1155.8 ± 52.0 g/2 rats/5 weeks, *P* > 0.05), body weights (Figure 1(a)), and body weight gain (Figure 1(b)). eWAT weight (Figure 1(c)), the ratio of eWAT weight to body weight (Figure 1(d)), and adipocyte size (Figures 1(f), 2(c), and 2(d)) tended to decrease, and adipocyte number (Figure 1(e)) tended to increase after ginger treatment. Basal plasma glucose and insulin concentrations, as well as the HOMA-IR index were decreased after treatment with 50 mg/kg ginger extract (Figures 3(a)–3(c)). During the OGTT assessment, the changes in plasma glucose concentrations (Figures 4(a) and 4(b)) were similar, but insulin concentrations (Figures 4(c) and 4(d)) were significantly lower in fructose ginger 50 mg/kg group than fructose-control group.

Ginger treatments did not significantly decrease basal plasma NEFA concentrations (Figure 5(a)), but fructose-induced increases in the Adipo-IR index (Figure 5(b)) and plasma NEFA concentrations (Figures 5(c) and 5(d)) during the OGTT assessment were reversed after treatment with ginger 50 mg/kg. 

### 3.3. Adipose Gene/Protein Expression Profiles in Rats

By real-time PCR, fructose-control rats showed significant increase in adipose *β*-actin, the house-keeping gene, compared to water-control rats. However, there was no difference in *β*-actin expression between fructose-control and fructose-ginger groups (data not shown). Thus, comparisons in gene expression are restricted to fructose-control and fructose-ginger groups.

**Figure 6:**
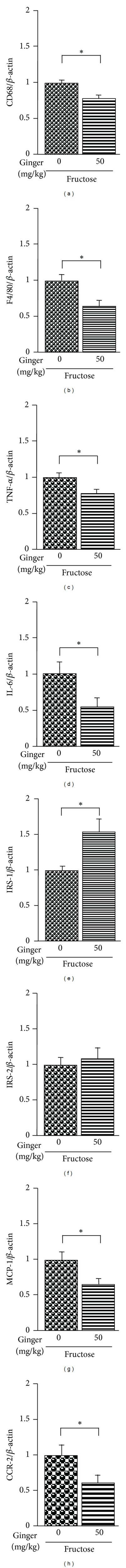
Adipose mRNA expression of CD68 (a), F4/80 (b), tumor necrosis factor (TNF)-*α* (c), interleukin (IL)-6 (d), insulin receptor substrates (IRS)-1 (e) and IRS-2 (f), monocyte chemotactic protein (MCP)-1 (g), chemokine (C-C motif) receptor (CCR)-2 (h) in fructose-pair-fed rats. The fructose-control rats had free access to 10% fructose in their drinking water, while the consumption of fructose in the ginger-(50 mg/kg) treated (by gavage daily) rats was adjusted to that of the fructose-control rats over 5 weeks. mRNA was determined by real-time PCR and normalized to *β*-actin. Levels in fructose control rats were arbitrarily assigned a value of 1. Data are means ± SEM (*n* = 6 each group). *P < 0.05.

Ginger treatment substantially suppressed adipose expression of CD68 (Figure 6(a)), F4/80 (Figure 6(b)), tumor necrosis factor (TNF)-*α* (Figure 6(c)), and interleukin (IL)-6 (Figure 6(d)). In contrast, expression of insulin receptor substrate (IRS)-1 (Figure 6(e)), but not IRS-2 (Figure 6(f)), was upregulated. Moreover, monocyte chemotactic protein (MCP)-1 (Figure 6 (g)) and chemokine (C-C motif) receptor-2 (CCR2) (Figure 6(h)) were also suppressed.

**Figure 7:**
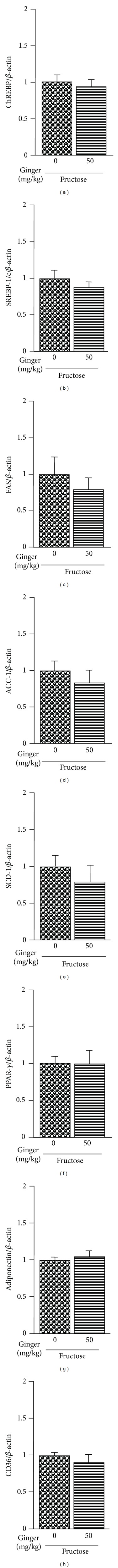
Adipose mRNA expression of carbohydrate-response-element-binding protein (ChREBP) (a), sterol-regulatory-element-binding protein (SREBP)-1c (b), fatty acid synthase (FAS) (c), acetyl-CoA carboxylase (ACC)-1 (d), stearoyl-CoA desaturase (SCD)-1 (e), peroxisome-proliferator-activated receptor (PPAR)-*γ* (f), adiponectin (g), and CD36 (h) in fructose-pair-fed rats. The fructose-control rats had free access to 10% fructose in their drinking water, while the consumption of fructose in the ginger-(50 mg/kg) treated (by gavage daily) rats was adjusted to that of the fructose-control rats over 5 weeks. mRNA was determined by real-time PCR and normalized to *β*-actin. Levels in fructose-control rats were arbitrarily assigned a value of 1. Data are means ± SEM (*n* = 6 each group). *P < 0.05.

In addition, ginger treatment had no significant effect on carbohydrate-response-element-binding protein (ChREBP) (Figure 7(a)), sterol-regulatory-element-binding protein (SREBP)-1c (Figure 7(b)), fatty acid synthase (FAS) (Figure 7(c)), acetyl-CoA carboxylase (ACC)-1 (Figure 7(d)), stearoyl-CoA desaturase (SCD)-1 (Figure 7(e)), peroxisome-proliferator-activated receptor (PPAR)-*γ* (Figure 7(f)), adiponectin (Figure 7(g)), and CD36 (Figure 7(h)).

## 4. Discussion

### 4.1. Ginger Treatment Improves Liquid-Fructose-Overconsumption-Induced Adipo-IR in Rats

 Insulin action in adipose tissue involves stimulation of glucose uptake and inhibition of lipolysis. However, adipose tissue only accounts for about 10% of insulin-stimulated glucose disposal [18]. In contrast, adipose tissue is the primary source of free fatty acids (~70%) for hepatic triglyceride synthesis [19]. In the setting of insulin resistance, insulin is unable to properly suppress lipolysis, resulting in an increase in free fatty acid release into the plasma [20]. Excess release of free fatty acids plays a key role in the development of lipotoxicity including liver injuries [6, 21–23]. Increased delivery of free fatty acids from adipose tissue leads to increases in fat accumulation, gluconeogenesis, and insulin resistance in liver [24, 25]. Therefore, plasma fatty acid changes during the OGTT assessment and the Adipo-IR index are used to evaluate insulin action in adipose tissues and analyze the contribution to the development of hepatic injuries including fatty liver [4–6, 9]. We have recently demonstrated that ginger treatment improves fructose-induced fatty liver in rats [16]. In the present study, long-term liquid fructose overconsumption increased the index of Adipo-IR and plasma NEFA and insulin concentrations at baseline and during the OGTT assessment in rats, indicating that fructose overconsumption induces Adipo-IR. The increases in Adipo-IR index and plasma concentrations of NEFA and insulin during the OGTT assessment were attenuated after treatment with ginger extract. These effects were accompanied by decrease in the HOMA-IR index, which reflects hepatic insulin sensitivity [5, 6]. Thus, these results suggest that ginger treatment improves fructose-induced Adipo-IR.

### 4.2. Suppression of Adipose Macrophage-Associated Proinflammatory Cytokines Contributes to Ginger-Treatment-Elicited Amelioration of Adipo-IR in Fructose-Fed Rats

 It is well known that inflammation in white adipose tissues (especially visceral fat) is associated with insulin resistance [1, 26, 27]. The macrophage accumulation in adipose tissue under an inflammatory state is a hallmark of obesity-induced insulin resistance, and the macrophages are responsible for almost all adipose tissue expression of TNF-*α* and IL-6, the markers of adipose macrophage polarization and inflammation [28, 29]. TNF-*α* is an important mediator of insulin resistance in obesity and diabetes through its ability to decrease the tyrosine kinase activity of the insulin receptor. TNF-*α* directly decreases insulin sensitivity and increases lipolysis in adipocytes [30, 31]. It has been demonstrated that treatment of cultured murine adipocytes with TNF-*α* induced serine phosphorylation of IRS-1 and converted IRS-1 into an inhibitor of the insulin receptor tyrosine kinase activity in vitro [32]. IL-6 plays a crucial role in metabolic processes and has adverse effects on insulin action in liver and adipose tissue [33]. IL-6 stimulates lipolysis to increase plasma NEFA concentrations in rats [34] and in human adipose tissue [35]. Insulin binding induces receptor tyrosine autophosphorylation, which is followed by the recruitment of scaffolding proteins known as IRS-1 and IRS-2 [18]. It has been suggested that IRS-1 works on the metabolism by regulating insulin signals in muscle and adipose tissues, whereas IRS-2 is a major player of hepatic insulin action [36]. IRS-1-deficient mice showed a phenotype of peripheral insulin resistance (mainly in muscle and white adipose tissue) [37, 38]. IL-6-mediated insulin resistance involves activation of proinflammatory kinases that converge at the IRS-1 level [39]. In the present study, 5-week fructose feeding tended to increase adiposity in rats, which was diminished by treatment with ginger extract. Importantly, ginger treatment suppressed adipose expression of CD68 and F4/80 (two important macrophage markers [29, 31]), indicating that ginger treatment decreases macrophage content in adipose tissue. Consistently, expression of the macrophage-associated proinflammatory cytokines, TNF-*α* and IL-6, was also downregulated. Further, the level of mRNA encoding IRS-1, but not IRS-2, was upregulated. Thus, these findings suggest that ginger treatment improves fructose-induced Adipo-IR via suppression of adipose macrophage accumulation-associated proinflammatory cytokines.

Macrophages may populate in adipose tissue during obesity through recruitment of chemokine-mediated chemotaxis. MCP-1 is an adipokine with insulin-resistance-inducing capacity that is related to increased adipose tissue mass in obesity and insulin resistance [40]. MCP-1 causes infiltration of macrophages to release proinflammatory proteins, such as TNF-*α* and IL-6 [30, 41, 42]. Studies have shown that overexpression of MCP-1 in adipose tissues causes macrophage recruitment and insulin resistance [30, 42]. In contrast, MCP-1 or its receptor CCR-2 knockout mice have fewer macrophages and less inflammation in adipose tissue and are protected from high-fat-diet-induced insulin resistance [41, 42]. Therefore, MCP-1 plays a pivotal role in the development of insulin resistance and is an important therapeutic target for improvement of insulin resistance [30, 31, 40–42]. In the present study, ginger treatment significantly suppressed adipose expression of MCP-1 and CCR-2 in fructose-fed rats, which was consistent with the downregulation of the adipose expression of macrophage-accumulation-mediated proinflammatory cytokines. Thus, it is likely that modulation of the adipose MCP-1-mediated pathway is involved in ginger-treatment-elicited suppression of the proinflammatory cytokines.

The constituents of ginger are numerous. Gingerols and shogaols (the latter is a dehydrated form of gingerols) are major components derived from ginger. [6]-gingerols and [6]-shogaol have been implicated in most of the pharmacological activities of ginger [43]. It has been reported that ginger components gingerols, [6]-shogaol and 1-dehydro-[10]-gingerdione, inhibit lipopolysaccharide-stimulated release and gene expression of proinflammatory cytokines including MCP-1 and IL-6 in RAW 264.7 macrophages or cultured primary rat astrocytes [44–47]. On the other hand, recent studies indicate that the recruited and activated neutrophils in adipose tissue produce chemokines and cytokines in response to excess energy intake [48, 49]. These neutrophils can facilitate macrophage infiltration, thereby contributing to the chronic low-grade-inflammation-associated insulin resistance [48, 49]. From here, thus, further investigation is needed to understand more about ginger detail by detail, such as the components responsible for the improvement of fructose-induced Adipo-IR, the specific function on macrophages, the manner in suppressing TNF-*α* and IL-6, and the role in modulating the functions of adipose neutrophils.

### 4.3. Ginger Treatment Does Not Alter Adipose PPAR-*γ*-, ChREBP-, and SREBP1c-Mediated Gene Expression in Fructose-Fed Rats

 PPAR-*γ* is a member of the ligand-activated nuclear receptor superfamily, expressed at high levels in adipose tissue; PPAR-*γ*-activating ligands improve adipose tissue function by altering fat topography and adipocyte phenotype and by upregulating genes encoding molecules that promote a combination of lipid storage and lipogenesis, such as CD36, SREBP-1, and SCD-1; PPAR-*γ* agonists promote the production of adiponectin in adipose tissue [8, 18]. Treatment with PPAR-*γ* agonist troglitazone upregulates the gene expression of PPAR-*γ*, ChREBP, and SREBP-1c in 3T3-L1 adipocytes [50]. It is known that ChREBP may play an important role in mediating de novo lipogenesis [12, 25]. However, the expression of ChREBP may be tissue dependent. Recent findings from humans have demonstrated that ChREBP mRNA and protein levels are increased in the liver from obese compared to lean subjects, whereas the expression is decreased in adipose tissues [51]. It has been reported that [6]-shogaol acts as a PPAR-*γ* agonist in 3T3-L1 adipocytes that originally derived from mice [52]. Recently, we have demonstrated that treatment with ginger extract substantially suppressed fructose-stimulated hepatic overexpression of the lipogenic protein/genes ChREBP, ACC-1, FAS, and SCD-1 in rats [16]. In the present study, however, treatment with ginger extract did not alter adipose mRNA levels of PPAR-*γ*, adiponectin, CD36, ChREBP, SREBP-1c, FAS, and ACC-1 in fructose-fed rats. Thus, our findings in gene expression do not support that the adipose PPAR-*γ*, ChREBP, and SREBP-1c pathways are involved in the improvement of Adipo-IR by ginger treatment. These results also suggest tissue-specific regulation of the lipogenic protein/genes by ginger treatment. Studies are needed to further clarify whether the difference in animal species (mice versus rats) and/or situation (in vitro versus in vivo) is associated with the discrepancy of the effect of ginger on PPAR-*γ*.

## 5. Conclusion

 The present results demonstrate that ginger treatment ameliorates fructose-overconsumption-induced adipose tissue insulin resistance in rats, which is associated with suppression of adipose macrophage-related proinflammatory cytokines. Our findings provide new insight into the pharmacological basis of therapeutics, especially for the traditional use of ginger in the prevention and treatment of metabolic derangements.

## Figures and Tables

**Table 1:** Primer sequences for real time PCR assays.

Gene	Forward primers	Reverse primers

*β*-actin	ACGGTCAGGTCATCACTATCG	GGCATAGAGGTCTTTACGGATG
ACC-1	AACATCCCGCACCTTCTTCTAC	CTTCCACAAACCAGCGTCTC
Adiponectin	CGTTCTCTTCACCTACGACCAGT	ATTGTTGTCCCCTTCCCCATAC
CCR-2	GAAGACCCAAAGACCAAGATGC	TCTGACAACAAAGCAGGAGGTG
CD36	AACCCAGAGGAAGTGGCAAAG	GACAGTGAAGGCTCAAAGATGG
CD68	ACTGGGGCTCTTGGAAACTACAC	CCTTGGTTTTGTTCGGGTTCA
ChREBP	TTGTTGGTGAGAAGTTCCGAAGG	CCCAGTAGAAGGGGTAAATGTTGAG
F4/80	ATCGCTGCTGGCTGAATACG	GCAACCTCGTATCCTTGAGCTTAG
FAS	ACCTCATCACTAGAAGCCACCAG	GTGGTACTTGGCCTTGGGTTTA
IL-6	GTTGCCTTCTTGGGACTGATGT	GGTCTGTTGTGGGTGGTATCCT
IRS-1	CTTCTGTTACACCTCAAGGGGC	GGTTATGGTTGGGACTTAGGTTCA
IRS-2	GACCAGTCCCACATCAGGCTT	CTGCACGGATGACCTTAGCG
MCP-1	CGGTTTCTCCCTTCTACTTCCTG	GCTCTGCCTCAGCCTTTTATTG
PPAR-*γ*	GCCCTTTGGTGACTTTATGGAG	GCAGCAGGTTGTCTTGGATGT
SCD-1	CAGTTCCTACACGACCACCACTA	GGACGGATGTCTTCTTCCAGAT
SREBP-1c	CTGTCGTCTACCATAAGCTGCAC	ATAGCATCTCCTGCACACTCAGC
TNF-*α*	ATGGGCTCCCTCTCATCAGTTC	CTCCTCCGCTTGGTGGTTTG

Sequences: 5′ to 3′.

## References

[1] Cornier MA, Dabelea D, Hernandez TL (2008). The metabolic syndrome. *Endocrine Reviews*.

[2] Boden G (1997). Role of fatty acids in the pathogenesis of insulin resistance and NIDDM. *Diabetes*.

[3] Attie AD, Scherer PE (2009). Adipocyte metabolism and obesity. *Journal of Lipid Research*.

[4] Gastaldelli A, Cusi K, Pettiti M (2007). Relationship between hepatic/visceral fat and hepatic insulin resistance in nondiabetic and type 2 diabetic subjects. *Gastroenterology*.

[5] Gastaldelli A, Harrison SA, Belfort-Aguilar R (2009). Importance of changes in adipose tissue insulin resistance to histological response during thiazolidinedione treatment of patients with nonalcoholic steatohepatitis. *Hepatology*.

[6] Neuschwander-Tetri BA (2010). Hepatic lipotoxicity and the pathogenesis of nonalcoholic steatohepatitis: the central role of nontriglyceride fatty acid metabolites. *Hepatology*.

[7] Lomonaco R, Ortiz-Lopez C, Orsak B (2012). Effect of adipose tissue insulin resistance on metabolic parameters and liver histology in obese patients with NAFLD. *Hepatology*.

[8] Sharma AM, Staels B (2007). Review: peroxisome proliferator-activated receptor *γ* and adipose tissue–understanding obesity-related changes in regulation of lipid and glucose metabolism. *Journal of Clinical Endocrinology and Metabolism*.

[9] Bell LN, Wang J, Muralidharan S (2012). Relationship between adipose tissue insulin resistance and liver histology in NASH: a PIVENS follow-up study. *Hepatology*.

[10] Johnson RJ, Perez-Pozo SE, Sautin YY (2009). Hypothesis: could excessive fructose intake and uric acid cause type 2 diabetes?. *Endocrine Reviews*.

[11] Stanhope KL, Schwarz JM, Keim NL (2009). Consuming fructose-sweetened, not glucose-sweetened, beverages increases visceral adiposity and lipids and decreases insulin sensitivity in overweight/obese humans. *The Journal of Clinical Investigation*.

[12] Tappy L, Le KA (2010). Metabolic effects of fructose and the worldwide increase in obesity. *Physiological Reviews*.

[13] Pollock NK, Bundy V, Kanto W (2012). Greater fructose consumption is associated with cardiometabolic risk markers and visceral adiposity in adolescents. *Journal of Nutrition*.

[14] Matsuda A, Wang Z, Takahashi S, Tokuda T, Miura N, Hasegawa J (2009). Upregulation of mRNA of retinoid binding protein and fatty acid binding protein by cholesterol enriched-diet and effect of ginger on lipid metabolism. *Life Sciences*.

[15] Nammi S, Kim MS, Gavande NS, Li GQ, Roufogalis BD (2010). Regulation of low-density lipoprotein receptor and 3-hydroxy-3- methylglutaryl coenzyme A reductase expression by zingiber officinale in the liver of high-fat diet-fed rats. *Basic and Clinical Pharmacology and Toxicology*.

[16] Gao H, Guan T, Li C (2012). Treatment with ginger ameliorates fructose-induced fatty liver and hypertriglyceridemia in rats: modulation of the hepatic carbohydrate response element binding protein-mediated pathway. *Evidence-Based Complementary and Alternative Medicine*.

[17] Rong X, Li Y, Ebihara K (2010). Angiotensin II type 1 receptor-independent beneficial effects of telmisartan on dietary-induced obesity, insulin resistance and fatty liver in mice. *Diabetologia*.

[18] Evans RM, Barish GD, Wang YX (2004). PPARs and the complex journey to obesity. *Nature Medicine*.

[19] Barrows BR, Parks EJ (2006). Contributions of different fatty acid sources to very low-density lipoprotein-triacylglycerol in the fasted and fed states. *Journal of Clinical Endocrinology and Metabolism*.

[20] Eckel RH, Grundy SM, Zimmet PZ (2005). The metabolic syndrome. *The Lancet*.

[21] Cusi K (2009). Role of insulin resistance and lipotoxicity in non-alcoholic steatohepatitis. *Clinics in Liver Disease*.

[22] Vanni E, Bugianesi E, Kotronen A, De Minicis S, Yki-Järvinen H, Svegliati-Baroni G (2010). From the metabolic syndrome to NAFLD or vice versa?. *Digestive and Liver Disease*.

[23] Fabbrini E, Sullivan S, Klein S (2010). Obesity and nonalcoholic fatty liver disease: biochemical, metabolic, and clinical implications. *Hepatology*.

[24] Bjorntorp P (1992). Metabolic abnormalities in visceral obesity. *Annals of Medicine*.

[25] Postic C, Girard J (2008). Contribution of de novo fatty acid synthesis to hepatic steatosis and insulin resistance: lessons from genetically engineered mice. *The Journal of Clinical Investigation*.

[26] Wellen KE, Hotamisligil GS (2005). Inflammation, stress, and diabetes. *The Journal of Clinical Investigation*.

[27] Ye J (2011). Adipose tissue vascularization: its role in chronic inflammation. *Current Diabetes Reports*.

[28] Weisberg SP, McCann D, Desai M, Rosenbaum M, Leibel RL, Ferrante AW (2003). Obesity is associated with macrophage accumulation in adipose tissue. *The Journal of Clinical Investigation*.

[29] Galic S, Fullerton MD, Schertzer JD (2011). AMPK *β*1 reduces mouse adipose tissue macrophage inflammation and insulin resistance in obesity. *The Journal of Clinical Investigation*.

[30] Kamei N, Tobe K, Suzuki R (2006). Overexpression of monocyte chemoattractant protein-1 in adipose tissues causes macrophage recruitment and insulin resistance. *The Journal of Biological Chemistry*.

[31] Di Gregorio GB, Yao-Borengasser A, Rasouli N (2005). Expression of CD68 and macrophage chemoattractant protein-1 genes in human adipose and muscle tissues: association with cytokine expression, insulin resistance, and reduction by pioglitazone. *Diabetes*.

[32] Hotamisligil GS, Peraldi P, Budavari A, Ellis R, White MF, Spiegelman BM (1996). IRS-1-mediated inhibition of insulin receptor tyrosine kinase activity in TNF-*α*- and obesity-induced insulin resistance. *Science*.

[33] Hoene M, Weigert C (2008). The role of interleukin-6 in insulin resistance, body fat distribution and energy balance. *Obesity Reviews*.

[34] Nonogaki K, Fuller GM, Fuentes NL (1995). Interleukin-6 stimulates hepatic triglyceride secretion in rats. *Endocrinology*.

[35] Trujillo ME, Sullivan S, Harten I, Schneider SH, Greenberg AS, Fried SK (2004). Interleukin-6 regulates human adipose tissue lipid metabolism and leptin production in vitro. *Journal of Clinical Endocrinology and Metabolism*.

[36] Biddinger SB, Kahn CR (2006). From mice to men: insights into the insulin resistance syndromes. *Annual Review of Physiology*.

[37] Araki E, Lipes MA, Patti ME (1994). Alternative pathway of insulin signalling in mice with targeted disruption of the IRS-1 gene. *Nature*.

[38] Tamemoto H, Kadowaki T, Tobe K (1994). Insulin resistance and growth retardation in mice made with targeted disruption of the IRS-1 gene. *Nature*.

[39] Benito M (2011). Tissue specificity on insulin action and resistance: past to recent mechanisms. *Acta Physiology*.

[40] Sell H, Eckel J (2007). Monocyte chemotactic protein-1 and its role in insulin resistance. *Current Opinion in Lipidology*.

[41] Kanda H, Tateya S, Tamori Y (2006). MCP-1 contributes to macrophage infiltration into adipose tissue, insulin resistance, and hepatic steatosis in obesity. *The Journal of Clinical Investigation*.

[42] Weisberg SP, Hunter D, Huber R (2006). CCR2 modulates inflammatory and metabolic effects of high-fat feeding. *Journal of Clinical Investigation*.

[43] Ali BH, Blunden G, Tanira MO, Nemmar A (2008). Some phytochemical, pharmacological and toxicological properties of ginger (*Zingiber officinale* Roscoe): a review of recent research. *Food and Chemical Toxicology*.

[44] Tripathi S, Maier KG, Bruch D, Kittur DS (2007). Effect of 6-gingerol on pro-inflammatory cytokine production and costimulatory molecule expression in murine peritoneal macrophages. *Journal of Surgical Research*.

[45] Dugasani S, Pichika MR, Nadarajah VD, Balijepalli MK, Tandra S, Korlakunta JN (2010). Comparative antioxidant and anti-inflammatory effects of [6]-gingerol, [8]-gingerol, [10]-gingerol and [6]-shogaol. *Journal of Ethnopharmacology*.

[46] Shim S, Kim S, Choi DS, Kwon YB, Kwon J (2011). Anti-inflammatory effects of [6]-shogaol: potential roles of HDAC inhibition and HSP70 induction. *Food Chemical Toxicology*.

[47] Lee HY, Park SH, Lee M (2012). 1-Dehydro-[10]-gingerdione from gingerinhibits IKK*β* activity for NF-*κ*B activation and suppresses NF-*κ*B-regulated expression of inflammatory genes. *British Journal of Pharmacology*.

[48] Kennedy A, Martinez K, Chuang CC, Lapoint K, Mcintosh M (2009). Saturated fatty acid-mediated inflammation and insulin resistance in adipose tissue: mechanisms of action and implications. *Journal of Nutrition*.

[49] Talukdar S, Da YO, Bandyopadhyay G (2012). Neutrophils mediate insulin resistance in mice fed a high-fat diet through secreted elastase. *Nature Medicine*.

[50] He Z, Jiang T, Wang Z, Levi M, Li J (2004). Modulation of carbohydrate response element-binding protein gene expression in 3T3-L1 adipocytes and rat adipose tissue. *American Journal of Physiology*.

[51] Hurtado del Pozo C, Vesperinas-García G, Rubio MÁ (2011). ChREBP expression in the liver, adipose tissue and differentiated preadipocytes in human obesity. *Biochimica et Biophysica Acta*.

[52] Isa Y, Miyakawa Y, Yanagisawa M (2008). 6-Shogaol and 6-gingerol, the pungent of ginger, inhibit TNF-*α* mediated downregulation of adiponectin expression via different mechanisms in 3T3-L1 adipocytes. *Biochemical and Biophysical Research Communications*.

